# Parental distress rating at the child’s age of 15 years predicts probable mental diagnosis: a three-year follow-up

**DOI:** 10.1186/s12887-022-03248-8

**Published:** 2022-04-04

**Authors:** Kristina Carlén, Sakari Suominen, Lilly Augustine, Maiju M. Saarinen, Minna Aromaa, Päivi Rautava, André Sourander, Matti Sillanpää

**Affiliations:** 1 School of Health Sciences, University of Skövde, Skövde, Box 408, 54128 Skövde, Sweden; 2 The Research School of Health and Welfare, Jönköping University, Jönköping, Sweden; 3 Department of Public Health, University of Turku, Turku, Finland; 4 CHILD, School of Learning and Communication, Jönköping University, Jönköping, Sweden; 5 Department of Child Neurology and General Practice, University of Turku and Turku University Hospital, Turku, Finland; 6 City of Turku Welfare Division, Turku, Finland; 7 Turku University Hospital, Clinical Research Centre, Turku, Finland; 8 Department of Child Psychiatry, University of Turku, Turku, Finland; 9 Department of Child Psychiatry, Turku University Hospital, Turku, Finland

**Keywords:** Assessment, Adolescents, Child Behavior Checklist, Mental health, Prediction

## Abstract

**Background:**

Mental health in adolescence is an increasing global public health concern. Over half of all mental disorders debut by 14 years of age and remain largely untreated up to adulthood, underlining the significance of early detection. The study aimed to investigate whether parental distress rating at the child’s age of 15 predicts a probable mental diagnosis in a three-year follow-up.

**Methods:**

All data was derived from the Finnish Family Competence (FFC) Study. The analysis focused on whether parental CBCL (Child Behavior Checklist) rating (*n* = 441) at the child’s age of 15 years predicted the outcome of the child’s standardised DAWBA (Development and Well-Being Assessment) interview at offspring’s 18 years.

**Results:**

Multivariable analysis showed that a one-unit increase in the total CBCL scores increased the relative risk of a DAWBA-based diagnosis by 3% (RR [95% CI] 1.03 [1.02–1.04], *p* < 0.001).

**Conclusions:**

Parental CBCL rating in a community sample at the adolescent’s age of 15 contributes to early identification of adolescents potentially at risk and thus benefitting from early interventions.

## Background

Mental health in adolescence is an increasing global public health concern. Perceived mental distress in youth can be seen as potential early signs of later psychiatric problems. The worldwide-pooled prevalence of mental disorders was 13.4% (CI 95% 11.3–15.9) in all age groups combined [1]. Between 15–19 years, combined prevalence is 14.1% for boys and 13.9% for girls for all mental disorders. Western Europe showed the highest gender prevalence difference worldwide among adolescents; 17% among girls and 16.1% among boys. In the distribution of mental disorders, anxiety and depressive disorders are the most common ones (42.9%), followed by conduct disorder (20.1%) and ADHD (19.5%) [2]. The World Health Organization (WHO) has estimated that over half of total cases of mental illness, including developmental disorders, begin by the age of 14 years, of which most remain untreated into adulthood [3]. As different internalising and externalising symptoms are common, early identification of these symptoms is crucial [4]. Therefore, this study focuses on symptoms of internalising (e.g., depression and anxiety) and externalising (e.g., conduct disorders). Parental assessment at the child’s age of 15 years could represent early detection of internal and external problems. Simultaneously, adolescents’ health is a priority area, as it even affects the next generation [5].

Some methods of detecting and identifying of mental disorders have been developed, for clinical use, whereas others are designed for unselected populations [6]. Identifying of mental health problems among an unselected population could be a critical tool in preparing and planning interventions, given that sufficient sensitivity is reached. As perceived mental health problems debut early, addressing them promptly and efficiently is vital before they potentially turn into clinical problems. To get a complete picture, many empirical scales collect data from several informants, such as self-reports, parents, teachers, and health services personnel. However, this cannot always be achieved, and only one informant is frequently referred to. The Child Behavior Checklist (CBCL), a parental assessment scale, has become a widely used instrument to identify child and adolescent emotional and behavioural problems in the community [7]. Parental ratings have demonstrated validity, especially concerning externalising problems [8]. A cross-sectional study from Italy [9] demonstrated the risk of mental disease being higher among children with parents of low socioeconomic status and level of education. Some studies have used instruments to explore the correlation between the prevalence of psychological problems and various psychopathologies at an early age, for example, Frigerio et al. [9]. In contrast, others have focused on factors associated with children’s mental-, and psychosocial well-being and functioning [10].

According to a national follow-up cohort, adolescent’s mental health problems predict future psychopathology and mental distress [11]. Some studies have targeted mental health problems in adolescents and the relationship with subsequent mental disorders in clinical settings according to the ICD-10 classification [6]. Others have compared the predictive power of parental and self-reported data for adolescents to validate different assessment scores [12]. However, the parental CBCL rating has not been studied to predict the subsequent adolescent risk of a mental disorder determined by a standardised Development and Well-Being Assessment (DAWBA) interview. Accordingly, the present study aimed to investigate the association of parental rating of their adolescent’s perceived mental distress and the risk of the adolescent’s probable mental health diagnosis three years later. Following the primary aim, the study naturally also thrives to contribute to the validity of parental reporting as an indicator of the subsequent mental health of their offspring.

## Methods

### Data collection

The present study used data from the ongoing Finnish Family Competence (FFC) study, based on a stratified randomised cluster sample from the Province of Turku and Pori in Southwestern Finland [13]. The study’s population originally consisted of families expecting their first baby and, therefore, paying their first visit to a maternity health care clinic in 1986. Earlier studies have described the sampling procedure in detail [13]. A total of 1287 babies born in the study area were included in the initial FFC study. At 12 years of age, 1125 (87%) children with their families still participated. At the adolescent’s age of 15 years, the CBCL questionnaires, one per family, were mailed home. Parents of 738 (66%) participating offspring returned the completed CBCL questionnaire in sealed envelopes (88% mothers, 8% fathers and 4% others, 9% single-parent families). At 18 years, 599 (53%) adolescents participated in a DAWBA interview. The present study consisted of 441 persons who showed data on both CBCL and DAWBA. Parents gave informed consent, and from 18 years, the adolescents did as well. The Joint Ethics Committee of the University of Turku and the Turku University Central Hospital approved the Finnish Family Competence Study design.

### Instruments

#### Child Behavior Checklist

CBCL is an assessment tool for parents rating adolescents’ behavioural, emotional, and social problems and competencies [14]. It is a widely used questionnaire addressing children’s and adolescents’ behavioural and emotional problems from 4–18 years, yielding a useable sum score [15]. The CBCL scale is a part of the Achenbach System of Empirically Based Assessment (ASEBA) and assesses language development and problem prevalence. This version of the CBCL includes two subscales, internalising and externalising scales and a total scale. The items can also be divided into eight syndrome scales: withdrawn, somatic complaints, anxious/depressed, social problems, thought problems, attention problems, delinquency, and aggressive behaviour [14]. In this study, the total scale was used. The parents rated 113 items concerning their child, such as ‘feels or complains that no one loves him/her’, ‘impulsive or acts without thinking’ or ‘cannot sit still, restless or hyperactive’. Each item related to ‘now’ or ‘within the past six months’ using a three-point scale: (0) Not true, (1) Sometimes/Somewhat True and (2) Very true/Often True. The possible score range was between 0–226. At least 93 of the 113 items had to be answered to be included in the study. The CBCL score is a validated scale used globally [15–17]. For total problems, scores <60 are considered in the normal range, scores >63 as clinical, and the range 60–63 represents a borderline [14]. Scores below the 95th percentile is considered being in the normal range, and above the 98th percentile is considered clinically notable [15]. Hence, an increase in the CBCL score indicates a higher prevalence of mental health problems even when these have not reached a clinically detectable level.

#### Development and Well-Being Assessment Scale

The DAWBA interview measures the risk of probable psychiatric diagnoses [18]. The DAWBA interview is designed to detect those at high risk for ICD-10 and DSV-IV psychiatric diagnoses in children and adolescents. This epidemiological tool can be clinically used but is predominately applied in research [19]. An increase in the DAWBA symptoms indicates a greater probability of a mental diagnosis, called a DAWBA-based diagnosis. It has been validated for predicting mental health disorders according to ICD-10 [6]. The DAWBA interview has shown good validity and is a well-documented tool for use both in the community and in clinical settings, kappa coefficient in a study was .64 [18]. In a randomised trial, the reliability of the interview was satisfactory. The agreement was significant for internalising disorders, 77% vs 68% for the randomly disclosed sample in relation to diagnosis according to ICD-10 compared to the not disclosed. For externalising disorders, the corresponding figures were 63% vs 71% [19]. DAWBA based-diagnosis has shown at least reasonably strong associations with both the ICD-10 and DSM-IV systems, though the DAWBA interview diagnosed more probable comorbid disorders [18].

DAWBA, a structured interview questionnaire, focuses on emotional (internalising), behavioural and hyperactivity (externalising) disorders. The structured interview is computerised and conducted by a professional interviewer with specific training in delivering the questions. In the present study, the interviewer was a specialised psychiatric nurse. The adolescents were invited to the interview by phone. After completing the interviews, the interviewer categorised all responses and valued based on computerised suggestions as a probable DAWBA-based diagnosis or not. All commenced interviews could be closed according to the protocol with no drop-outs or incomplete responses. Possible DAWBA-based diagnoses were social phobia, agoraphobia, panic disorders, autism spectrum disorders, post-traumatic stress disorder, obsessive-compulsive disorder, generalised anxiety disorder, depression disorders, conduct disorders and eating disorders (www.dawba.info). The Finnish version was retranslated into English for consultation with Goodman, the author of the scale [18].

#### Background variables

As background variables, the child’s gender (female vs. male), parents’ age at child’s birth, parents’ educational level (≤ 9 years vs. >9 years), parents’ additional vocational education (≤ vocational school vs. college/university) were included. In Finland, all children attend a 9-year primary mandatory education. Parents’ socioeconomic status (SES) was measured according to their professional status (blue-collar vs. white-collar).

### Statistical methods

Univariate and multivariable modified Poisson regression analyses were used for binary data [20]. The outcome variable comprised a dichotomous variable, ‘any DAWBA-based diagnosis vs. no diagnosis at the adolescents’18 years of age. Background variables chosen from previous research [9, 21] were included as covariates. The principal explanatory variable was the CBCL total sum score as a continuous variable at the child’s age of 15. The covariates’ associations with DAWBA were checked using single predictor models. If significant, the variables were included in the initial multivariable models (inclusion criteria *p* < 0.1). In this step, the model also included pairwise interactions of covariates with CBCL to find whether the association between CBCL and DAWBA would change the covariate values. As no significant interactions were found, only main effects were included in the final multivariable models. The association between DAWBA and CBCL remained after adjusting for these covariates. All non-significant *p*-values (*p* < 0.05) were excluded from the final multivariable model using backward selection. Cases with missing data in CBCL were excluded from the analyses (*n* = 158). The results are presented as a risk ratio (RR) with a 95% confidence interval (CI). Based on a drop-out analysis of all included covariates, those declining a DAWBA interview were more often boys and children of mothers with lower education. Regarding CBCL, the only statistically significant difference concerned the slightly lower proportion of mothers having reached at least college as their vocational education in the group that declined to respond to the CBCL items. ROC analyses investigated the 90th and 95th percentile of CBCL scores’ distribution and analysed cut-off values. Statistical computations used the SAS System for Windows, Release 9.4 (SAS Institute, Cary, NC, USA).

## Results

Most of the total participants (*n* = 441) were girls (57%; *n* = 251), and a substantial majority of them (85%; *n* = 214) did not report symptoms in the DAWBA interview (see Table 1). A total of 15% (*n* = 37) girls and 5% (*n* = 10) boys did receive a DAWBA-based diagnosis.

**Table 1:** Descriptive statistics of adolescents according to DAWBA, preceding parental CBCL, and baseline data

	No DAWBA-based diagnosis (No Dg) ***n*** = 394	DAWBA-based diagnosis (Dg) ***n*** = 47	Difference between means* Dg – no Dg	***p***-value
**CBCL**	**Median (IQR)** **Mean (SD)**	**Median (IQR)** **Mean (SD)**		
CBCL score at the age of 15 years	10.6 (15.0)	21.0 (23.2)	4.22	<.001
	14.8 (13.5)	24.4 (16.5)		
**Gender**	**% (n)**	**% (n)**		
Girls (*n* = 251)	54.3 (214)	78.7 (37)	10.20	.001
Boys (*n* = 190)	45.7 (180)	21.3 (10)		
**Parental data at child’s birth**	**Median (IQR)** **Mean (SD)**	**Median (IQR)** **Mean (SD)**		
Mother’s age (total *n* = 433)	26.6 (4.9)	25.3 (5.2)	2.60	.010
	27.2 (3.7)	25.5 (4.1)		
Father’s age (total *n* = 407)	29.0 (5.1)	28.1 (5.2)	1.84	ns
	29.5 (4.2)	28.0 (4.9)		
	**% (n)**	**% (n)**		
Living in single parent families at 15 years (*n* = 53)	11.4 (45)	17.0 (8)		
Mother’s basic education >9 years(*n* = 232)	53.1 (209)	49.0 (23)	0.28	ns
<9 years(*n* = 209)	47.0 (185)	51.1 (24)		
Father’s basic education >9 years (*n* = 139)	31.5 (124)	31.9 (15)	0.00	ns
<9 years (*n* = 302)	68.5 (270)	68.1 (32)		
Mother’s vocational training				
college/university (*n* = 187)	42.9 (169)	38.3 (18)	0.36	ns
< college/university (*n* = 254)	57.1 (225)	61.7 (29)		
Father’s vocational training				
college/university (*n* = 132)	30.7 (121)	23.4 (11)	1.07	ns
< college/university (*n* = 309)	69.3 (273)	76.6 (36)		
Mother’s SES white-collar (*n* = 47)	11.2 (44)	6.4 (3)	1.01	ns
<white-collar (*n* = 394)	88.8 (350)	93.6 (44)		
Father’s SES white-collar (*n* = 112)	26.7 (105)	14.9 (7)	3.06	ns
<white-collar (*n* = 329)	73.4 (289)	85.1 (40)		

*Group differences are tested using Wilcoxon Two-Sample Test and Chi-Square

Descriptive statistics, mean differences, and *p*-values of two groups from ratings according to the Development and Well-Being Assessment (DAWBA) Scale of 18-year-olds and gender inclusive parental data at the child’s birth, and the Child Behavior Checklist (CBCL) at age 15 (*n* = 441)

**Fig. 1:**
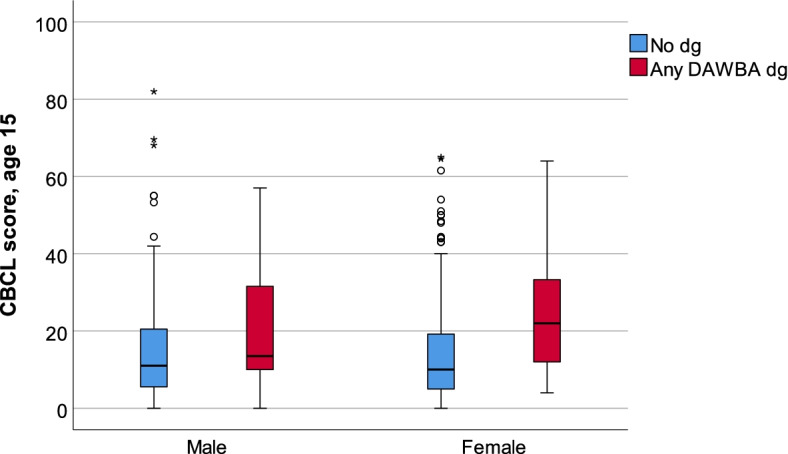
The distribution of CBCL scores for gender (male (*n* = 10), female (*n* = 37)) and DAWBA-based diagnosis (No diagnosis, any DAWBA diagnosis)

The CBCL scores at the age of 15 differed according to whether the adolescent developed a DAWBA-based diagnosis at 18 years of age (Median = 21.0 IQR = 23.2) vs no diagnosis (Median = 10.6 IQR = 15.0). The range of the total CBCL scores was 0–82 in the group with no symptoms and 0–64 in the DAWBA-based diagnosis group (see Fig. 1).

The most common diagnoses were depression (28%) and social anxiety (21%). The CBCL scores at the 90th percentile were 34, which included 9.5% (*n* = 42) of adolescents with a subsequent DAWBA-based diagnosis; of these, 33% (*n* = 14) were boys and 67% girls (*n* = 28). The corresponding values for the 95th percentile were 47, 4.9% (*n* = 22), 36% (*n* = 8) boys and 64% (*n* = 14) girls. Using a clinical cut-off (>60 points) included only one girl (0.2%) and none of the boys. The AUC value for the ROC analysis of all the participants of this study is 0.69 and according to gender for boys 0.61 and girls 0.71, respectively.

Regarding background factors: The mother’s age at the child’s birth varied from 19.1–39.8 in the group with no symptoms (Median = 26.6 IQR = 4.9), vs. 17.2–34.9, (Median = 25.3 IQR = 5.2) in the DAWBA-based diagnosis group. For the father’s age, the variation ranged from 19.3–48.8 (Median = 28.1 IQR = 5.2) in the group with no symptoms vs 19.7–40.1 (Median = 29.0 IQR = 5.1) in the group with a DAWBA-based diagnosis. Parents had lower education in the group of those who received a diagnosis at the age of 18 years. In the sample, 9% (*n* = 53) lived in a single-parent family at 15 years of age, of which 17% (*n* = 8) developed a DAWBA-based diagnosis. The distribution of the CBCL scores, gender, and other background variables according to DAWBA-based groups are given in Table 1, and the distribution of the CBCL scores according to gender is illustrated in Fig. 1.

In Table 2, the risk ratios for receiving a DAWBA-based diagnosis are presented. In the multivariable analysis, a one-unit increase in CBCL increased the relative risk of a DAWBA-based diagnosis by 3%. Thus, each additional difficulty or an increased frequency of a reported difficulty, rated by the parent, increased the likelihood of a future mental health diagnosis by 3%. Female adolescents had nearly a threefold risk, and a one-year decrease in the father’s age was related to an increased relative risk of a DAWBA-based diagnosis by 9%.

**Table 2:** Risk ratios for a DAWBA-based diagnosis of mental disorders among adolescents at 18 years

	Univariate			Multivariable		
	RR	95% CI	p	RR	95% CI	p
CBCL at age 15 1-unit increase	1.03	1.02–1.04	<0.001	1.03	1.02–1.04	<0.001
Gender, female vs. male	2.80	1.43–5.49	0.003	2.85	1.49–5.47	0.002
Father’s age, 1-year decrease	1.08	0.99–1.18	0.082	1.09	1.00–1.19	0.041
Mother’s age, 1-year decrease	1.12	1.02–1.23	0.014			
Father’s SES, blue-collar vs. higher	1.95	0.90–4.23	0.092			

## Discussion

Our follow-up study showed that a raise by one-unit in the CBCL total score is significantly associated with an increased relative risk of a DAWBA-based diagnosis by more than 3% three years later. Theoretically, an increase by circa three points would probably imply one more diagnosis among 100 adolescents than a corresponding group with in average of three points less. The value of the CBCL lies in its non-clinical nature, as the parents can notice trends before others do [18]. Therefore, non-clinical values on the CBCL score is related to the increased risk of a later diagnosis by 3%.

The results confirmed our hypothesis that the parental assessment of adolescents’ mental health problems could predict probable mental diagnoses among adolescents. One additional unit of the CBCL scale indicates the adolescent having one additional problem area or having a more severe problem within a particular area. The mean CBCL difference between those identified by a probable DAWBA-based diagnosis and those that were not was 10 units, with few clinical scores (>60). Only one received a probable diagnosis three years later of those who did get a clinical score (*n* = 7). Using the cut-off above 60 in the CBCL [14] was too high to be applied in this population. Using the 95th percentile as cut-off identified 47 adolescents above the level of which only seven (14.9%) received a subsequent DAWBA-based diagnosis. However, we found that using the 90th percentile (CBCL score 34), ten adolescents received a DAWBA-based diagnosis (21.2%). This cut-off score of the CBCL might be a value for finding those at risk of developing a psychiatric diagnosis in three years of follow-up, with a sensitivity of 24% and a specificity of 93% at the 90th percentile. This means that the study method contributes to the identification of adolescents in need of closer follow-up from the age of 15 years onwards more than using CBCL as a screening tool for finding all at risk. The AUC values for identifying adolescents using the CBCL tool was better than chance (>0.5), it was.69. The predictability was better for girls than for boys (.71 vs. .61). However, in a community population, the clinically useful cut-off values consequently must be set lower, but nevertheless, parental ratings still predicted to some extent the subsequent mental health prospects of the adolescents. To the best of our knowledge, no previous study has focused on the prevalence of mental health problems and subsequent mental disorders in an epidemiological community setting using concomitantly the two clinically validated assessment instruments CBCL and DAWBA. The CBCL scale is used for studying children’s and adolescents’ perceived distress, predominantly in clinical and community settings [8]. A longitudinal study from the Netherlands of a community-based sample [21] investigated trajectories of psychosocial problems, with CBCL as the outcome variable, in adolescents 11, 14 and 17 years of age and showed that early well-child clinic assessments predicted the outcome. The assessments comprised evaluations from parents, from well-child clinic professional, and well-child records. Low parental educational level and single parenthood predicted emotional problems for both boys and girls [21].

DAWBA was initially created for assessing children’s and adolescents’ psychopathology as a supporting tool for the planning of services [18]. One study investigating the independent correlates of a probable DAWBA-based diagnosis in a community sample of 491 adolescents showed ORs between 8 and 27, depending on the informant. The lowest OR for a diagnosis was observed when adolescents self-reported and the highest in cases when mental health care was provided. The DAWBA interview represents an assessment method of mental health that can potentially be applied even in clinical, not only in community settings [18] which supports the validity of parental assessments [18]. Aebi and colleagues [12] compared the contribution of parent and youth data contribution using DAWBA and Strengths and difficulties questionnaire (SDQ) to identify adolescents’ mental disorders or mental health problems. They found that parent reports alone are sometimes reasonable substitutes for screening purposes if one cannot obtain information from multiple sources like teachers, youth or healthcare services [12]. The study indicates that parental reports, at least on a group level, show that screening can be conducted based on single informants if additional information is difficult to obtain.

Girls have been found to have more emotional problems than boys [21, 22], which is also reflected in the present study’s findings, according to which more girls than boys were identified to have higher scores of CBCL. Still, the boys had more extreme outliers (see Fig. 1). CBCL and DAWBA measure more than emotional problems, such as externalising issues, which are more common in boys [23]. However, in our study, girls were three times more likely to receive a diagnosis according to DAWBA than boys. The threshold for revealing emotional problems seems to be lower for girls and they talk more about such kinds of problems than boys [24]. The greater risk of girls having a mental diagnosis might partially be related to parents’ more sensitive recognition of internalising difficulties, not only to the higher prevalence in girls [12]. This is in line with previous findings according to which access to information from the parents increased the ability to detect adolescents’ emotional problems in the community [25]. Conversely, parents should see externalising symptoms more easily than internalising symptoms, such as depression [12].

We also found that one year’s decrease in the father’s age at the child’s birth was significantly related to an increased relative risk of a DAWBA-based diagnosis by 9%.The univariate analysis in Table 2 shows that the unadjusted risk for young mothers was higher, but the association disappeared in the multivariable analysis. However, a young mother only indicated the mother being inexperienced, and it is more common for women than men to have relations with older partners [26]. Two young parents, rather than just one, are probably responsible for why the mother’s age was not included in the final multivariable analysis. Thus, in the concurrent Finnish context, a young father indicated in most cases also a young mother, which equals two young parents. A longitudinal study from Denmark focusing on individuals born in 1955 to 2006 and followed from 1995 to 2011 showed that, in contrast, young mothers in combination with old fathers are associated with an increased risk of mental disorders in their offspring due to biological and sociocultural factors [27]. However, a Swedish national register study found that elderly fathers’ children were born in a wealthier and healthier context [28]. Accordingly, the high age of the father often means a better economy, which indirectly could be protective for the offspring in several aspects [26].

A limitation of the study is the inevitable attrition during the total follow-up time of 18 years. At the beginning of the 1980ies, a sample of more than 1000 children and families was considered very large. The double recruitment process, i.e. consent to participation from the parents, had to be confirmed by the adolescents after they reached the age of 18, which may have decreased the response rate. A longitudinal study needing this double recruitment process may consider a 35% response rate of theoretically available observations satisfactory. A drop-out analysis showed that more boys and children whose mothers had lower education declined the DAWBA interview. Regarding decline to respond to the CBCL items, only mothers’ lower educational level significantly differed decliners from participants. Therefore, in the end, a small health selection for the benefit of those better off might have taken place. This health selection has likely underestimated the predictive power of CBCL scores because they are more likely to be looking at a healthy population. According to the authors’ interpretation, it does not entitle the conclusion that the main findings would have been caused by selection. Due to the small sample size, any manipulation might profoundly affect the results. As well, additional data collections between 15 years and 18 years would have been helpful. Nevertheless, we think that the design is strong enough to justify the actual conclusions, as the limitation most probably has not been biased but only potentially weakened the present results. Finally, from a methodological perspective, the closeness of the relation between parent and child is probably also reflected in the reliability of the CBCL score. Hence, difficulties in the relationship between parents, which in itself is a problem and a risk for subsequent mental health deterioration of the child, might also be reflected in the selective inaccuracy of parental reporting.

This study also has strengths; longitudinal epidemiological data collection extends from birth until 18 years of age. The prospective design based on an original population-based sample allows early detection of mental health problems when the family visited the healthcare clinic, during school age and even during the transition to adulthood, for all 18 years. We had compared variables gathered when the child was born and measured aspects of parental ratings at adolescents’ age of 15 years and finally about the prevalence of a probable mental diagnosis at the age of 18 years for adolescents. Secondly, the original sample is population-based, including all newborns in a particular geographic area. Thus, our study well represents adolescents in a concurrent modern Western society. Thirdly, DAWBA represents an applicable contributory method to arrive at a proposed mental diagnosis in a population-based setting. The most significant strength is the knowledge that CBCL can serve as an early indicator of the risk of a subsequent mental health diagnosis in adolescents.

## Conclusions

Parental CBCL rating, even in a community setting, is a contributory tool for identifying potentially increased risk of offspring’s subsequent mental diagnoses at the child’s age of 15 years. Moreover, the CBCL rating contributes to identifying adolescents that need monitoring more closely and potentially benefit most from early interventions in school settings or at health care clinics.

## Data Availability

The data the study is based on contains personal and intimate information that according to initial agreements with the participants from the start of the follow-up cannot be transferred to third parties or outside the borders of Finland. Theoretically some subsets of data could on reasonable request be delivered but unfortunately, in most cases it probably would be evaluated as not possible along with this. The datasets used and analysed during the current study are available from the corresponding author on reasonable request.

## Abbreviations

ALSPAC: Avon Longitudinal Study of Parents and Children
ASEBA: Achenbach System of Empirically Based Assessment
CBCL: Child Behavior Check List
DAWBA: Development and Well-Being Assessment
DSV-IV: Diagnostic and Statistical Manual of Mental Disorders, 4th Edition
FFC: Finnish Family Competence
ICD-10: International Classification of Diseases, 10th Revision
WHO: World Health Organization
